# A bibliometric analysis of the top 100 most cited papers and research trends in breast cancer related *BRCA1* and *BRCA2* genes

**DOI:** 10.1097/MD.0000000000030576

**Published:** 2022-09-23

**Authors:** Shadi Alkhayyat, Muhammad Khan, Tauseef Ahmad, Haroon, Huma Tariq, Mukhtiar Baig

**Affiliations:** a Department of Medicine, Faculty of Medicine, King Abdulaziz University Hospital, King Abdulaziz University, Jeddah, Saudi Arabia; b Department of Biotechnology and Genetic Engineering, Hazara University Mansehra, KP, Pakistan; c Vanke School of Public Health, Tsinghua University, Beijing, China; d Department of Epidemiology and Health Statistics, School of Public Health, Southeast University, Nanjing, China; e College of Life Science, Northwest University, Xian, China; f Department of Zoology, Hazara University Mansehra, KP, Pakistan; g Department of Clinical Biochemistry, Faculty of Medicine, Rabigh, King Abdulaziz University, Jeddah, Saudi Arabia.

**Keywords:** bibliometric analysis, *BRCA1*, *BRCA2*, breast cancer

## Abstract

This study aimed to identify, characterize, and map the important attributes of the top 100 most cited papers on *BRCA1* and *BRCA2* genes. The scientific literature on *BRCA1* and *BRCA2* was searched in the Web of Science Core Collection database using the keywords “*BRCA1*” OR “*BRCA2*” (Title). The top 100 most cited papers were selected based on citations. The obtained data were exported into HistCite^TM^, RStudio, and VOSviewer software for prerequisite analysis. The top 100 most cited papers on *BRCA1* and *BRCA2* were authored by 932 authors from 24 countries and published in 27 journals. These papers were cited 79,713 times, ranging from 441 to 4671 citations. The highly cited paper was cited 4671 times and published in *Science* (1994). The leading author, journal, publication year, institution, and country were Easton DF (n = 16), *Nature Genetics* (n = 11), 2002 (n = 11), University of Pennsylvania (n = 17), and the USA (n = 76), respectively. The results show that all the top 100 papers were produced in developed countries. The collaboration index among the authors was 9.49. The most frequently appeared keywords were ovarian-cancer, breast-cancer, mutations, gene, and familial breast. In recent times, the trend topics were patients, mutations, carriers, ovarian, and risk.

## 1. Introduction

Breast cancer, the most frequently diagnosed and reported cause of death, threatens women’s health in many countries.^[1]^ Certain factors play an important role in protecting against breast cancer development, including late menarche and more nursing duration.^[2–4]^ Ovarian hormone exposure, the number of ovulatory cycles, and breast lobular differentiation are suggested to reduce the occurrence of breast cancer.^[5–7]^ Researchers have evaluated the effects of reproductive factors on *BRCA*-related breast cancer, but the results are conflicting and differ by mutations either in *BRCA1* or *BRCA2*.^[8–10]^ Few studies suggest no impact of breastfeeding on breast cancer development, but few have reported its protective effect on breast cancer risk in *BRCA1* carriers.^[11–13]^ Breastfeeding did not show any impact on breast cancer risk in *BRCA2* mutation carriers.^[10]^

In 2020, 2.3 million women were diagnosed with breast cancer, and it caused 685,000 deaths worldwide. At the end of 2020, 7.8 million women living with breast cancer ranked it the most prevalent cancer globally.^[14]^ Breast cancer and recent trends in its research are topics of prime importance.^[15]^ The importance of quantitative and qualitative assessment of the available scientific literature in this field has increased. Such assessments can play a key role in funding concerning research projects and resource priority setting, as demonstrated in the Research Assessment Exercise.^[16]^

The bibliometric analysis introduces a review process that allows the published research to be better described, evaluated, and monitored. It covers quantitative and visual processes in scientific publications to define their characteristics and dynamics. Bibliometric studies are either evaluation or relational studies. Evaluation studies estimate the number of cited papers and total citations. Relational studies talk about the structure of a research issue, research profiling of authors, institutional affiliations, new research avenues, and scientific techniques employed. Appropriate interpretation is very important in bibliometric studies.^[17]^ The present study aims to identify and analyze the top 100 most cited papers in *BRCA1* and *BRCA2* associated breast and ovarian cancers worldwide using comprehensive data analysis and bibliometric tools. Furthermore, this study can help researchers and policymakers devise policies regarding the diagnosis and treatment of breast and ovarian cancers.

## 2. Methods

### 2.1. Study design

A descriptive bibliometric study was conducted.

### 2.2. Search strategy and database

**Figure 1.**
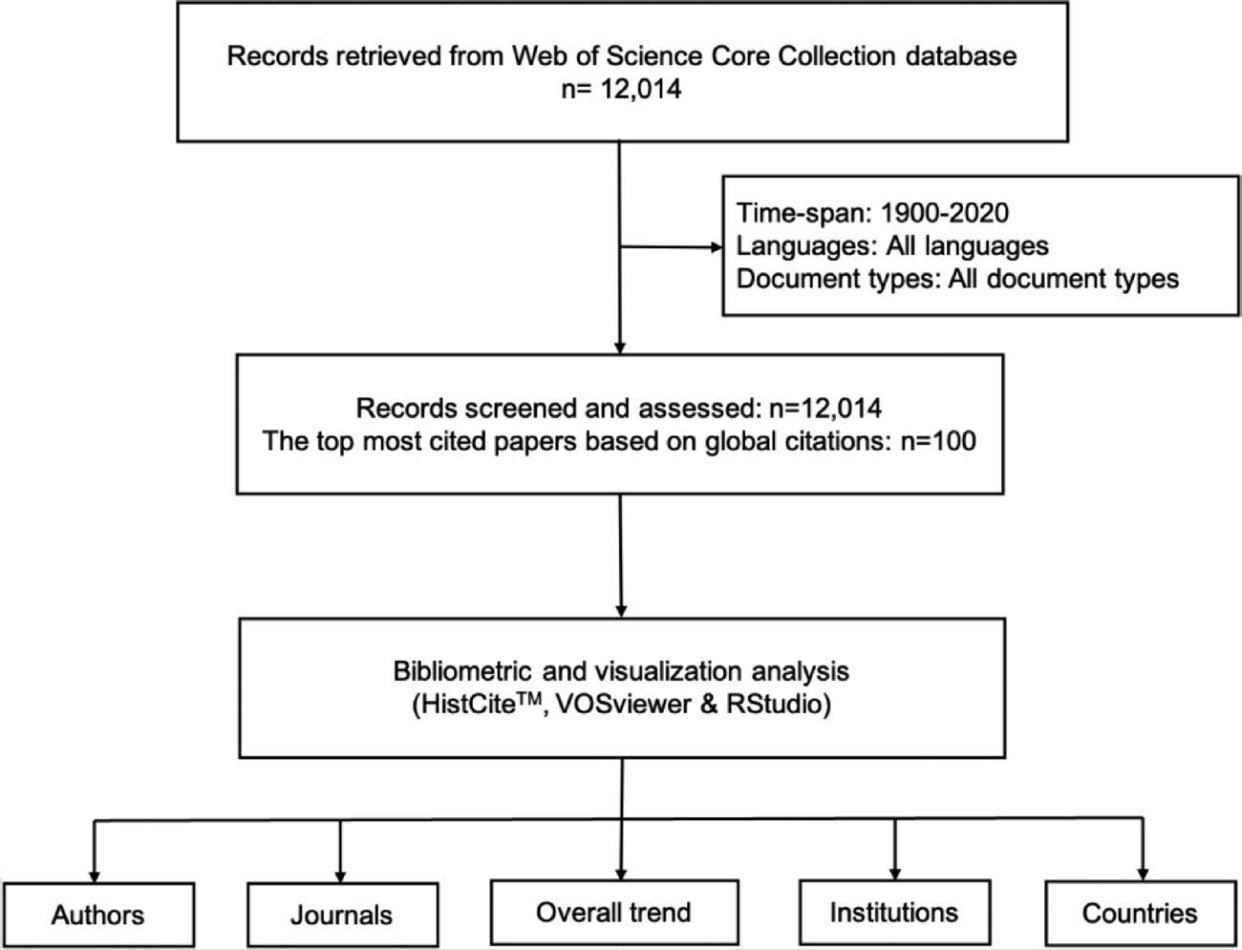
Study flow chart.

A comprehensive review and discussion regarding the topic took place before conducting the online search to decide the retrieval database and potential search keywords. On June 07, 2021, the online search was performed through the Web of Science Core Collection (WoSCC) database hosted by Clarivate Analytics (https://clarivate.com/webofsciencegroup/solutions/web-of-science/), previously the Intellectual Property and Science business of Thomson Reuters, Philadelphia, Pennsylvania, USA (Fig. 1).

The WoSCC database is the world’s oldest database.^[18]^ Previously published studies in different research fields utilized WoSCC databases.^[19–27]^ The WoSCC database was accessed through the online library portal of Southeast University, Nanjing, China (http://www.lib.seu.edu.cn/). The potential search keywords were entered in the database search engine using Boolean search operators “*BRCA1*” OR “*BRCA2*” in the title field without restrictions.

### 2.3. Selection of publications

Researchers in this study independently screened the retrieved documents to identify the top 100 most cited publications on *BRCA1*- and *BRCA2*-related breast and ovarian cancer. The disagreements were resolved after a discussion with the third researcher. Only publications that focused on *BRCA1* and *BRCA2* causing breast and ovarian cancer were included in the final analysis. The top 100 most cited publications were identified based on the number of global citation score (GCS) in descending order, and publications that had more citations were ranked higher.

### 2.4. Extraction of data

A self-designed electronic data form was designed for data collection. After selecting the top 100 most cited publications, major key attributes were extracted from the data. For this purpose, the data were exported into Microsoft Excel 2019 to extract the required data and calculate the frequencies and percentages of the included publications. The following information was extracted: study title, year of publication, journal, authors name, institution, country, publication types, publication language, widely used keywords, and number of citations. The journals’ impact factor (IF) and quartile ranking (Q1–4) were obtained from Incites Journal Citation Reports 2020, released in June 2021 by Clarivate Analytics. The data were downloaded in 2 formats: plain text and comma-separated values.

### 2.5. Data analysis

For data analysis, 3 different tools were used. OriginPro 2018 software was used to generate the relevant graphs. For citation analysis, the data were exported in to HistCite^TM^ software.^[28]^ Furthermore, the data were plotted for visualization networks using VOSviewer software version 1.6.17 for windows.^[29]^ In addition, RStudio (biblioshiny package) was used to generate the cloud map, countries’ collaboration, and trend analysis.

### 2.6. Ethics and consent

This study involved no animal and human subjects; therefore, no ethical approval was required.

## 3. Results

### 3.1. General characteristics

The top 100 most cited papers on *BRCA1* and *BRCA2* were authored by 932 authors from 24 countries and published in 27 journals. These papers were published between 1994 and 2017. The GCS of these papers was 79,713 (797.1 citations per paper), ranging from 441 to 4671 citations. However, the overall local citation score was 588. The top 100 most cited papers were published in English, of which 93 papers were published as research articles. The overall collaboration among the authors was 9.49. The main facts about the top 100 most cited papers are presented in Table 1.

**Table 1:** Main facts about the top 100 most cited papers.

Description	Results
Main information	
Time-span	1994–2017
Documents	100
Publishing language (English)	100
Journals	27
Institutions	292
Countries	24
Average years from publication	20
Average citations per documents	797.1
Average citations per year per documents	41.23
Global citations score	79,713
Local citations score	588
References	2545
Document types	
Article	93
Review	4
Letter	3
Document contents	
Keywords Plus	411
Author’s keywords	23
Authors	
Authors	932
Author appearances	1324
Authors of single-authored documents	2
Authors of multi-authored documents	930
Author collaboration	
Single-authored documents	2
Documents per author	0.107
Authors per document	9.32
Co-authors per documents	13.2
Collaboration index	9.49

### 3.2. Top 10 highly cited papers

The highly cited paper was “A strong candidate for the breast and ovarian cancer susceptibility gene *BRCA1*” published by Miki et al (1994) in *Science* and was cited 4671 times. The top 10 most cited papers along with their conclusion bottom lines can be seen in Table 2, while the top 100 cited papers can be found in supplementary data (Table S1, Supplemental Digital Content 1, http://links.lww.com/MD/H319). Of the top 100 most cited papers, 15 papers were cited more than 1000 times, 58 papers from 500 to 999 times, and 27 papers from 441 to 499 times.

**Table 2:** The top 10 most cited publications with basic information and bottom lines.

Ranking	Study title [reference]	GCS	GCS per year	Bottom lines
1	A strong candidate for the breast and ovarian-cancer susceptibility gene BRCA1^[30]^	4671	194.63	BRCA1 was identified as a potential candidate gene implicated in breast and ovarian cancer if mutated. Pathogenic insertions, deletion, substitutions, and regulatory mutations were identified in this study in different kindred for breast and ovarian cancer progression
2	Specific killing of BRCA2-deficient tumors’ with inhibitors of poly (ADP-ribose) polymerase^[31]^	2894	222.62	BRCA2 mutations damage the self-repair mechanism of DNA, which usually enables the DNA to make necessary repairs after certain breakdowns. PARP inhibitors play an important role in identifying a deficiency in homologous recombination due to deficient BRCA2 mutant cells
3	Identification of the breast cancer susceptibility gene BRCA2^[32]^	2501	108.74	BRCA2 was identified as a potential gene implicated in breast cancer
4	Average risks of breast and ovarian cancer associated with BRCA1 or BRCA2 mutations detected in case series unselected for family history: a combined analysis of 22 studies^[33]^	2304	153.60	A meta-analysis was performed to estimate breast or ovarian cancer risks associated with different BRCA1 and BRCA2 mutations
5	Genetic heterogeneity and penetrance analysis of the BRCA1 and BRCA2 genes in breast cancer families^[34]^	2144	107.20	The contribution of BRCA1 and BRCA2 for inherited cases of breast cancer was assessed in 237 families, each with at least 4 cases of breast cancer. The disease was associated with BRCA1 in 52% of families, BRCA2 in 32% of families, and neither of these in 16% of families. Penetrance estimates were also provided in the study, which can be used for better counselling of mutation carriers
6	The risk of cancer associated with specific mutations ofBRCA1 and BRCA2 among Ashkenazi Jews^[35]^	1635	77.86	Cancer risk of 3 specific mutations of BRCA1 and BRCA2 was estimated among Ashkenazi Jews
7	Breast and ovarian cancer risks due to inherited mutations in BRCA1 and BRCA2^[36]^	1477	98.47	Breast and ovarian cancer risks were estimated among Ashkenazi Jewish women carrying inherited mutations in BRCA1 and BRCA2
8	Risks of cancer in BRCA1-mutation carriers^[37]^	1429	59.54	The study estimated the risk of breast and ovarian cancer from the occurrence of second cancer. There is a greater chance of getting ovarian cancer if an individual is suffering from breast cancer and vice versa
9	Localization of a breast cancer susceptibility gene, BRCA2, to chromosome 13Q12-13^[38]^	1402	58.42	Genetic reason for breast cancer was identified in families with cancer unlinked to the BRCA1 locus. Implication of BRCA2, a new gene on chromosome 13q1 2-13, was identified. BRCA2 confers a high risk of breast cancer but does not confer a substantially higher risk for ovarian cancer
10	Oral poly (ADP-ribose) polymerase inhibitor olaparib in patients with BRCA1 or BRCA2 mutations and advanced breast cancer: a proof-of-concept trial.^[39]^	1261	157.63	A concept trial of ADP-ribose inhibitor, Olaparib was carried out in patients with BRCA1 and BRCA2 mutations detected. This inhibition showed a favorable therapeutic index for targeted treatment in breast cancer patients from Australia, Germany, Spain, Sweden, UK, and the USA

GCS = global citation score.

### 3.3. Year of publications and citations score

**Figure 2.**
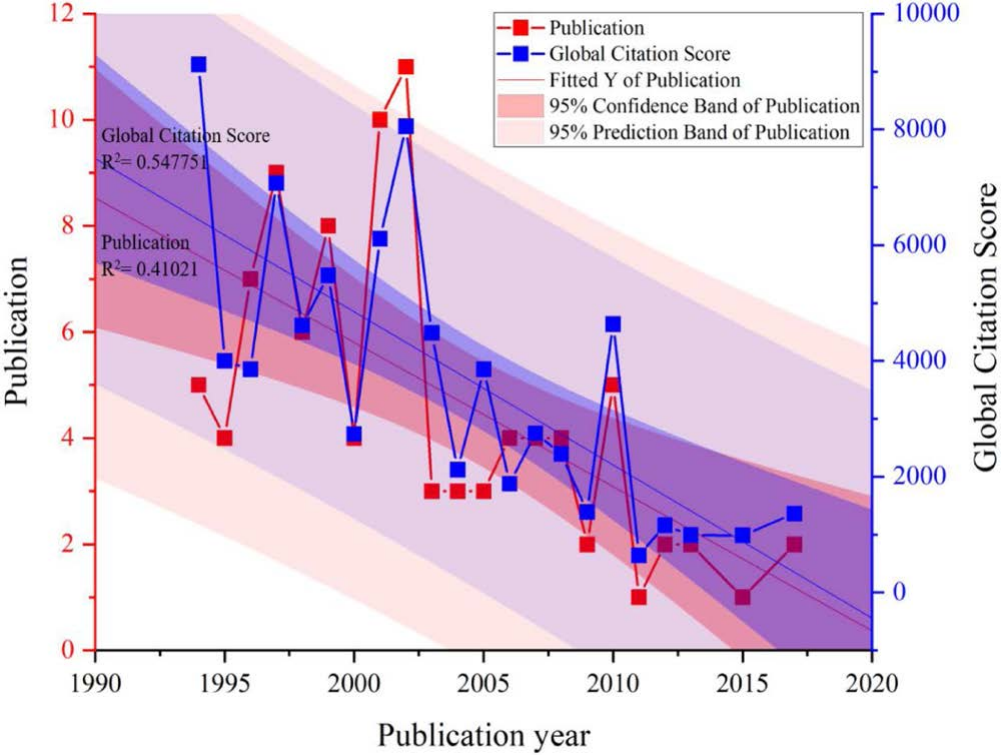
Year of publication and global citation score.

The most productive years were 2002 (n = 11), and 2001 (n = 10), while the most cited years were 1994 (n = 9124), and 2002 (n = 8054). In total, 56% of the most cited papers were published between 1994 and 2002. The annual scientific publication growth rate was found to be −4.27%. As shown in Figure 2, the coefficient of determination (*R*^2^) of published papers was 0.4.

### 3.4. Leading authors, journals, institutions, and countries

The most prolific authors were Easton DF (n = 16) and Peto J (n = 11), while the most cited authors were Easton DF (n = 16,176) and Narod S (n = 14,422) as shown in Table 3. *Nature Genetics* (n = 11), and *Science* (n = 10) were the top-ranked journals, while the most cited journals were *Science* (n = 12,095), and *Nature* (n = 8752). The *New England Journal of Medicine* had the highest IF (91.253) as shown in Table 4.

**Table 3:** Authors with at least 5 publications.

Authors	Publications	LCS	LCS per year	GCS	GCS per year	Local cited references
Easton DF	16	121	5.9	16,176	1632.47	71
Peto J	11	115	5.39	13,022	669.58	47
Devilee P	8	96	4.31	9672	464.98	29
Stratton MR	8	60	2.69	7361	389.89	33
Ford D	7	101	4.53	8870	399.65	24
Livingston DM	7	65	3.35	4481	264.42	55
Narod S	7	118	5.37	14,422	699.43	21
Narod SA	7	44	2.36	5726	353.64	54
Ponder BAJ	7	112	5.11	8919	404.99	27
Weber BL	7	34	1.89	4426	262.42	39
Couch FJ	6	26	1.66	3487	289.8	46
Goldgar D	6	96	4.26	9970	456.3	25
Rebbeck TR	6	37	2.19	5692	412.87	38
Bishop DT	5	79	3.6	7025	321.98	22
Evans DG	5	11	0.93	5049	462.05	30
Garber JE	5	16	1.18	4349	400.9	33
Haites N	5	53	2.47	4994	249.39	26
Jasin M	5	30	1.9	3281	306.74	37
Lenoir G	5	61	2.86	6221	290.84	24
Lynch H	5	86	3.81	8101	362.31	9
Neuhausen S	5	91	4.11	8431	378.55	26
Seal S	5	48	2.1	5940	307.24	13
Weber B	5	68	3.15	5189	243.46	24

GCS = global citation score, LCS = local citation score.

**Table 4:** The top journals with at least 5 publications.

Journals	Publications	Citations	Citations per year	IF 2020 (5-year)	Quartile ranking (category rank)
*Nature Genetics*	11	6447	344.58	38.33 (36.431)	Q1 (2/176)
*Science*	10	12,095	630.29	47.728 (51.434)	Q1 (2/72)
*Molecular Cell*	9	5587	391.46	17.97 (19.639)	Q1 (6/295)
*Cell*	8	6185	448.11	41.584 (46.899)	Q1 (2/295)
*Journal of The National Cancer Institute*	8	4797	373.17	13.506 (13.893)	Q1 (16/242)
*Nature*	8	8752	565.37	49.962 (54.537)	Q1 (1/72)
*JAMA-Journal of The American Medical Association*	7	4470	896.76	56.274 (60.151)	Q1 (3/167)
*American Journal of Human Genetics*	5	6798	375.77	11.025 (12.095)	Q1 (11/176)
*Journal of Clinical Oncology*	5	3838	522.85	44.544 (33.883)	Q1 (4/242)
*New England Journal of Medicine*	5	4666	257.27	91.253 (89.676)	Q1 (1/167)

IF = impact factor, Q = quartile.

The institution with maximum published papers was the University of Pennsylvania (n = 17), followed by Memorial Sloan Kettering Cancer Centre (n = 15), and the University of Cambridge (n = 15). However, the most cited institutions were the University of Cambridge (n = 16,069), the University of Utah (n = 15,252), and the University of Pennsylvania (n = 12,792), as shown in Table 5. As shown in Table 6, the USA was the most productive and cited country (n = 76, citations = 62,105), followed by the UK (n = 32, citations = 30,970), and Canada (n = 20, citations = 24,112).

**Table 5:** Institutions with at least 5 publications.

Institutions	Publications	LCS	GCS
Univ Penn	17	87	12,792
Mem Sloan Kettering Canc Ctr	15	84	11,960
Univ Cambridge	15	122	16,069
Dana Farber Canc Inst	14	38	10,194
Inst Canc Res	12	98	12,348
Univ Utah	12	155	15,252
Int Agcy Res Canc	10	113	10,956
Netherlands Canc Inst	10	29	6735
Harvard Univ	9	35	5231
NCI	9	60	7052
Creighton Univ	8	88	9705
Leiden Univ	8	96	9672
Yale Univ	8	67	7743
McGill Univ	7	116	12,655
St Marys Hosp	7	21	6767
Univ Texas	7	57	4071
Univ Toronto	7	50	5977
Univ Aberdeen	6	54	5477
Univ Melbourne	6	21	5830
Womens Coll Hosp	6	48	6352
Addenbrookes Hosp	5	55	4427
Fox Chase Canc Ctr	5	19	3901
Imperial Canc Res Fund	5	80	7070
Inst Curie	5	48	4486
Massachusetts Gen Hosp	5	9	3080

GCS = global citation score, LCS = local citation score.

**Table 6:** Countries with at least in 5 publications.

Countries	Publications	LCS	GCS
USA	76	493	62,105
UK	32	189	30,970
Canada	20	197	24,112
Netherlands	16	96	14,664
France	12	95	9265
Australia	10	25	9168
Germany	8	16	6050
Sweden	7	58	11,627
Spain	6	8	4874
Iceland	5	74	6261
Ireland	5	30	3306

GCS = global citation score, LCS = local citation score.

### 3.5. Keywords and trend topics analysis

**Figure 3.**
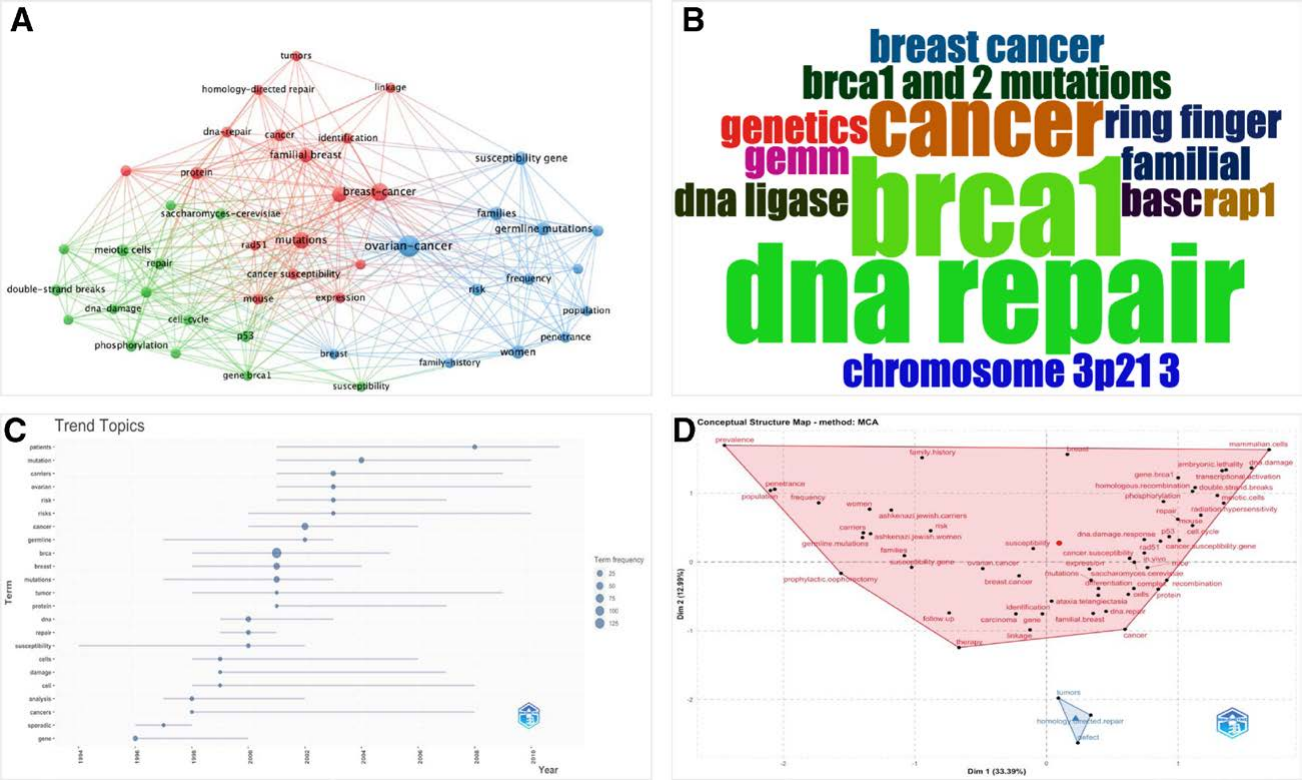
(A) KeyWords Plus network visualization mapping, (B) WordCloud map of author’s keywords, (C) trend topics analysis, and (D) factorial analysis (conceptual structure map).

The minimum number of occurrences of a keyword was fixed at 5, while the minimum cluster size was 10. Of the total KeyWords Plus (n = 411), only 45 met the threshold, and were plotted into 3 clusters, and each color represented a different cluster; red color (cluster 1, n = 17), green color (cluster 2, n = 15), and blue color (cluster 3, 13), as shown in Figure 3A. The most frequently appearing KeyWords Plus were ovarian-cancer (n = 31), breast-cancer (n = 20), mutations (n = 16), gene (n = 12), familial breast (n = 11), families (n = 10), germline mutations (n = 10), susceptibility gene (n = 10), women (n = 10), and protein (n = 9). However, the frequently used author’s keywords were BRCA1 (n = 3), DNA repair (n = 3), and cancer (n = 2), as shown in Figure 3B. In recent times, the trend topics based on title were patients, mutations, carriers, ovarian, and risk, as shown in Figure 3C. The factorial analysis map was generated based on multiple correspondence analysis method as shown in Figure 3D. The field was selected as KeyWords Plus. The data were plotted into 2 clusters; cluster 1 (red color) and cluster 2 (blue color). Cluster 1 is grouped by ovarian cancer, breast cancer, mutations, gene, familial breast, families, germline mutations, and other topics related to cancer, women, risk, protein, expressions, identification, in vivo, linkage, carcinoma, prevalence, recombination, and therapy. Cluster 2 is grouped by homology directed repair, tumors, and defect.

### 3.6. Corresponding author’s country and collaboration world map

**Figure 4.**
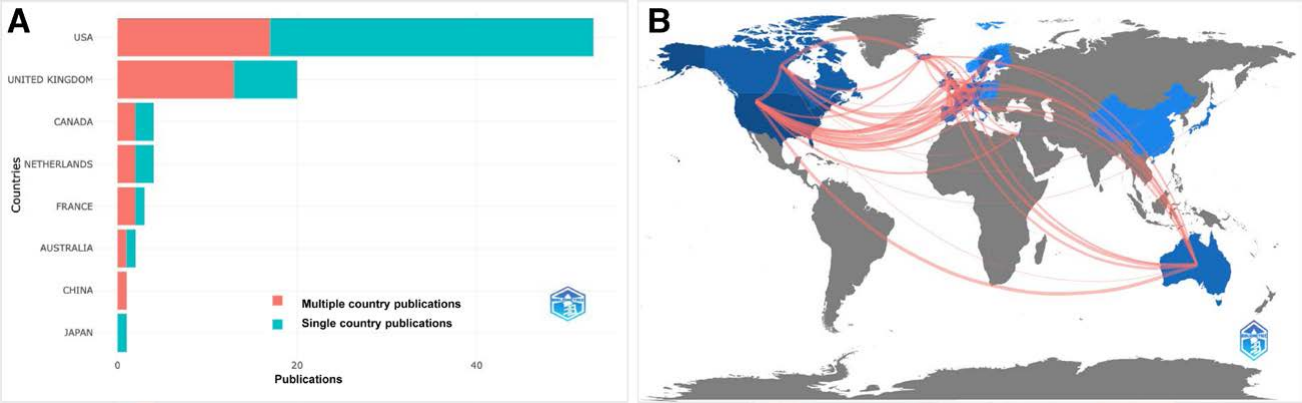
(A) Corresponding author’s country, (B) collaboration world map.

The USA had the highest multiple country and single country corresponding author publications, 17 and 36, respectively, as shown in Figure 4A. As shown in Figure 4B, the USA had the strongest collaboration with the UK (n = 16), followed by the UK and the Netherlands (n = 15), the USA and Netherlands (n = 12), the UK and France (n = 11), and the USA and Canada (n = 11).

## 4. Discussion

This study is the first comprehensive bibliometric analysis of the top 100 most cited papers on *BRCA1 and BRCA2* associated breast and ovarian cancer globally. Each year, 1.3 million breast cancer cases are diagnosed globally, which is the most common invasive cancer in females.^[40]^ *BRCA1* and *BRCA2* are among the most common genes implicated in the progression of breast and ovarian cancer. Therefore, it is important for the scientific community to identify and study the most influential papers in the field. This analysis can help fulfill the missing gaps, provide the recent and potential trends in this area, and direct attention to the critical aspects that have not been addressed yet. The outcomes of this study include the most prolific authors, most published journals, most active institutions, highly contributing countries, keywords and trend topics analysis, and citations count.

In our analysis, the most published journal was *Nature Genetics* which published 11 articles, while *Science* published 10, and *New England Journal of Medicine* published only 5 articles among the top 100 cited articles. The IF of *Nature Genetics* is the lowest compared to the other 2 journals. This phenomenon indicates that the relevancy of a journal to the nature of study plays a vital role than the IF of a journal.

In this study, the overall GCS was 79,713 (ranging from 441 to 4671 citations), while the local citation score was 588 (ranging from 1 to 43 citations). In the top 100 most cited papers, 5 papers were cited more than 2000 times (2144–4671 citations), 10 papers were cited more than 1000 times (1014–1635 citations), 58 papers were cited at least 500 times (500–986 citations), while the remaining papers (n = 27) were cited more than 400 times (441–494 citations).

Generally, papers published in relevant and reputed journals with important public health issues attract more readers and can be cited multiple times. In the current study, no smooth ascending or descending trend in the year-wise citations was observed. This variation may be due to the current trends in the field, as the *BRCA* study tends more to the diagnosis while treatment is a crucial step after the diagnosis. Scientists may have focused on other aspects of the disease.

The included studies were mainly based on experimental work, that is, research articles (93%). This indicates that the research emphasis has been directed toward diagnosis, treatment, and applications rather than literature-based work. All the 100 top-cited papers were published in English, which is the leading scientific language used globally for research dissemination.

The University of Pennsylvania was the top leading institution in terms of publications, while the most cited institution was the University of Cambridge. In this category, universities remained on top while hospitals and centers remained on the bottom. This trend highlights the understandable fact of different objectives of universities and hospitals.

Among the countries involved in the top 100 cited papers, the USA was the leading country in terms of publications, citations, and collaboration. This trend is in line with many other studies in various research fields.^[41–50]^ None of the resource-limited countries were involved in the highly influential studies as the main contributor or leader. This trend has various reasons, for which funding allocation by any country is the most important. In the USA, research and development is funded by a number of sectors, including the federal government, academia, businesses, nonprofit organizations, and state governments for a variety of purposes. As a result, in the 20th century, the USA has become a global leader in research and development.^[51–52]^

During the period between 2003 and 2007, studies were conducted on the topics related to patients, mutations, carriers, ovarian, and risk factors. However, an examination of the top 10 most cited studies indicates that 90% of the studies focused on diagnosis, while 10% focused on treatment. It is observed that after performing certain work on diagnosis, the scientist moved to treatment as the main aim of diagnosis is to use this information for treatment. This trend has shown a ray of hope that scientists have assessed this problem and are seriously working to solve it step by step.^[53]^ Another study found that GD2 could be used as a monitoring target in clinical isolates from breast cancer patients.^[54]^

This study will not only be important for medical practitioners but also for policymakers for better decisions. More research needs to be carried out in this field, and researchers, especially from disease burdened countries, should be encouraged to carry out research and share their findings in peer-reviewed journals.

## 5. Conclusion

This bibliometric study should boldly call out that the vast majority of the most influential papers originate from the USA, the UK, and Canada, clustering around well-known institutions. Interestingly, these contributions are well respected and deeply appreciated. However, it would benefit the community at large to have industry and individual government funding directed more towards high-quality research and peer-reviewed research publications in low-income countries.

The most frequently appearing keywords were ovarian-cancer, breast-cancer, mutations, gene, and familial breast. In recent times, the trend topics were patients, mutations, carriers, ovarian, and risk. A better understanding of breast and ovarian cancer’s clinical features along with screening of *BRCA1* and *BRCA2* in the community may lead to timely diagnosis and management. This can partially lead to a better quality of life for the patients and can help to reduce the occurrence of hereditary breast cancer in future generations.

## 6. Limitations

This study has some limitations. Firstly, only the WoSCC database was utilized. Secondly, the search was limited to the title field. Thirdly, the self-cations of the authors were not excluded. Therefore, the above limitations may bias the frequency of publications frequency and citation score.

## Author contributions

**Conceptualization:** Shadi Alkhayyat, Muhammad Khan, Tauseef Ahmad.

**Data curation:** Muhammad Khan, Tauseef Ahmad.

**Formal analysis:** Tauseef Ahmad.

**Methodology:** Shadi Alkhayyat, Muhammad Khan, Tauseef Ahmad.

**Project administration:** Tauseef Ahmad.

**Software:** Tauseef Ahmad.

**Validation:** Muhammad Khan, Tauseef Ahmad, Haroon, Mukhtiar Baig.

**Visualization:** Tauseef Ahmad.

**Writing – original draft:** Shadi Alkhayyat, Muhammad Khan, Tauseef Ahmad, Haroon, Huma Tariq.

**Writing – review & editing:** Muhammad Khan, Tauseef Ahmad, Huma Tariq, Mukhtiar Baig.

## Paper context

Breast cancer is the most frequently diagnosed and reported cause of death in women. To characterize important attributes of the top 100 most cited papers in breast and ovarian cancer-related BRCA1 and BRCA2 genes. Overall, the papers included in the current study were cited 79,713 times (ranging from 441 to 4671). The most published journal was Nature Genetics. The most productive country was the USA. However, collaborations with developed countries are needed to promote breast cancer research in developing countries.

## Supplementary Material


