# Supplementary material for: A bibliometric analysis of the top 100 most cited papers and research trends in breast cancer related BRCA1 and BRCA2 genes
Source: Medicine (Baltimore). 2022 Sep 23;101(38):e30576. doi: 10.1097/MD.0000000000030576 (PMC9509046; doi:10.1097/MD.0000000000030576)
Supplement: Supplementary file 1 [file medi-101-e30576-s001.pdf]

Supplementary Materials

(A bibliometric analysis of the top 100 most cited papers and research trends in breast cancer related BRCA1 and BRCA2 genes)

Supplementary Table 1: Top most cited papers on *BRCA1* and *BRCA2* genes

Ranking	References	Local citations score	Global citations score
1	MIKI Y, SWENSEN J, SHATTUCKEIDENS D, FUTREAL PA, HARSHMAN K, et al.<br>A STRONG CANDIDATE FOR THE BREAST AND OVARIAN-CANCER SUSCEPTIBILITY GENE BRCA1<br>SCIENCE. 1994 OCT 7; 266 (5182): 66-71	43	4671
2	Bryant HE, Schultz N, Thomas HD, Parker KM, Flower D, et al.<br>Specific killing of BRCA2-deficient tumours with inhibitors of poly(ADP-ribose) polymerase<br>NATURE. 2005 APR 14; 434 (7035): 913-917	10	2894
3	WOOSTER R, BIGNELL G, LANCASTER J, SWIFT S, SEAL S, et al.<br>IDENTIFICATION OF THE BREAST-CANCER SUSCEPTIBILITY GENE BRCA2<br>NATURE. 1995 DEC 21; 378 (6559): 789-792	26	2501
4	Antoniou A, Pharoah PDP, Narod S, Risch HA, Eyfjord JE, et al.<br>Average risks of breast and ovarian cancer associated with BRCA1 or BRCA2 mutations detected in case series unselected for family history: A combined analysis of 22 studies<br>AMERICAN JOURNAL OF HUMAN GENETICS. 2003 MAY; 72 (5): 1117-1130	9	2304
5	Ford D, Easton DF, Stratton M, Narod S, Goldgar D, et al.<br>Genetic heterogeneity and penetrance analysis of the BRCA1 and BRCA2 genes in breast cancer families<br>AMERICAN JOURNAL OF HUMAN GENETICS. 1998 MAR; 62 (3): 676-689	21	2144
6	Struewing JP, Hartge P, Wacholder S, Baker SM, Berlin M, et al.<br>The risk of cancer associated with specific mutations of BRCA1 and BRCA2 among Ashkenazi Jews<br>NEW ENGLAND JOURNAL OF MEDICINE. 1997 MAY 15; 336 (20): 1401-1408	20	1635
7	King MC, Marks JH, Mandell JB<br>Breast and ovarian cancer risks due to inherited mutations in BRCA1 and BRCA2<br>SCIENCE. 2003 OCT 24; 302 (5645): 643-646	6	1477
8	FORD D, EASTON DF, BISHOP DT, NAROD SA, GOLDGAR DE, et al.<br>RISKS OF CANCER IN BRCA1-MUTATION CARRIERS<br>LANCET. 1994 MAR 19; 343 (8899): 692-695	20	1429
9	WOOSTER R, NEUHAUSEN SL, MANGION J, QUIRK Y, FORD D, et al.<br>LOCALIZATION OF A BREAST-CANCER SUSCEPTIBILITY GENE, BRCA2, TO CHROMOSOME 13Q12-13<br>SCIENCE. 1994 SEP 30; 265 (5181): 2088-2090	17	1402
10	Tutt A, Robson M, Garber JE, Domchek SM, Audeh MW, et al.<br>Oral poly(ADP-ribose) polymerase inhibitor olaparib in patients with BRCA1 or BRCA2 mutations and advanced breast cancer: a proof-of-concept trial<br>LANCET. 2010 JUL 24; 376 (9737): 235-244	2	1261

11	Scully R, Chen JJ, Plug A, Xiao YH, Weaver D, et al.<br>Association of BRCA1 with Rad51 in mitotic and meiotic cells<br>CELL. 1997 JAN 24; 88 (2): 265-275	23	1238
12	Venkitaraman AR<br>Cancer susceptibility and the functions of BRCA1 and BRCA2<br>CELL. 2002 JAN 25; 108 (2): 171-182	9	1198
13	Chen SN, Parmigiani G<br>Meta-analysis of BRCA1 and BRCA2 penetrance<br>JOURNAL OF CLINICAL ONCOLOGY. 2007 APR 10; 25 (11): 1329-1333	2	1074
14	FUTREAL PA, LIU QY, SHATTUCKEIDENS D, COCHRAN C, HARSHMAN K, et al.<br>BRCA1 MUTATIONS IN PRIMARY BREAST AND OVARIAN CARCINOMAS<br>SCIENCE. 1994 OCT 7; 266 (5182): 120-122	14	1069
15	Rebbeck TR, Lynch HT, Neuhausen SL, Narod SA, van't Veer L, et al.<br>Prophylactic oophorectomy in carriers of BRCA1 or BRCA2 mutations<br>NEW ENGLAND JOURNAL OF MEDICINE. 2002 MAY 23; 346 (21): 1616-1622	8	1014
16	Kaufman B, Shapira-Frommer R, Schmutzler RK, Audeh MW, Friedlander M, et al.<br>Olaparib Monotherapy in Patients With Advanced Cancer and a Germline BRCA1/2 Mutation<br>JOURNAL OF CLINICAL ONCOLOGY. 2015 JAN 20; 33 (3): 244-250	0	986
17	Bunting SF, Callen E, Wong N, Chen HT, Polato F, et al.<br>53BP1 Inhibits Homologous Recombination in Brca1-Deficient Cells by Blocking Resection of DNA Breaks<br>CELL. 2010 APR 16; 141 (2): 243-254	3	975
18	Audeh MW, Carmichael J, Penson RT, Friedlander M, Powell B, et al.<br>Oral poly(ADP-ribose) polymerase inhibitor olaparib in patients with BRCA1 or BRCA2 mutations and recurrent ovarian cancer: a proof-of-concept trial<br>LANCET. 2010 JUL 24; 376 (9737): 245-251	2	969
19	Wang Y, Cortez D, Yazdi P, Neff N, Elledge SJ, et al.<br>BASC, a super complex of BRCA1-associated proteins involved in the recognition and repair of aberrant DNA structures<br>GENES & DEVELOPMENT. 2000 APR 15; 14 (8): 927-939	7	954
20	Cancer risks in BRCA2 mutation carriers<br>JNCI-JOURNAL OF THE NATIONAL CANCER INSTITUTE. 1999 AUG 4; 91 (15): 1310-1316	0	949
21	Moynahan ME, Chiu JW, Koller BH, Jasin M<br>Brca1 controls homology-directed DNA repair<br>MOLECULAR CELL. 1999 OCT; 4 (4): 511-518	13	944
22	Garcia-Higuera I, Taniguchi T, Ganesan S, Meyn MS, Timmers C, et al.<br>Interaction of the fanconi anemia proteins and BRCA1 in a common pathway<br>MOLECULAR CELL. 2001 FEB; 7 (2): 249-262	0	907
23	Kauff ND, Satagopan JM, Robson ME, Scheuer L, Hensley M, et al.<br>Risk-reducing salpingo-oophorectomy in women with a BRCA1 or BRCA2 mutation.<br>NEW ENGLAND JOURNAL OF MEDICINE. 2002 MAY 23; 346 (21): 1609-1615	6	890

24	Lim E, Vaillant F, Wu D, Forrest NC, Pal B, et al.<br>Aberrant luminal progenitors as the candidate target population for basal tumor development in BRCA1 mutation carriers<br>NATURE MEDICINE. 2009 AUG; 15 (8): 907-913	0	863
25	Howlett NG, Taniguchi T, Olson S, Cox B, Waisfisz Q, et al.<br>Biallelic inactivation of BRCA2 in Fanconi anemia<br>SCIENCE. 2002 JUL 26; 297 (5581): 606-609	5	846
26	Sharan SK, Morimatsu M, Albrecht U, Lim DS, Regel E, et al.<br>Embryonic lethality and radiation hypersensitivity mediated by Rad51 in mice lacking Brca2<br>NATURE. 1997 APR 24; 386 (6627): 804-810	18	840
27	Esteller M, Silva JM, Dominguez G, Bonilla F, Matias-Guiu X, et al.<br>Promoter hypermethylation and BRCA1 inactivation in sporadic breast and ovarian tumors<br>JNCI-JOURNAL OF THE NATIONAL CANCER INSTITUTE. 2000 APR 5; 92 (7): 564-569	3	836
28	Domchek SM, Friebel TM, Singer CF, Evans DG, Lynch HT, et al.<br>Association of Risk-Reducing Surgery in BRCA1 or BRCA2 Mutation Carriers With Cancer Risk and Mortality<br>JAMA-JOURNAL OF THE AMERICAN MEDICAL ASSOCIATION. 2010 SEP 1; 304 (9): 967-975	1	828
29	Warner E, Plewes DB, Hill KA, Causer PA, Zubovits JT, et al.<br>Surveillance of BRCA1 and BRCA2 mutation carriers with magnetic resonance imaging, ultrasound, mammography, and clinical breast examination<br>JAMA-JOURNAL OF THE AMERICAN MEDICAL ASSOCIATION. 2004 SEP 15; 292 (11): 1317-1325	1	794
30	Cortez D, Wang Y, Qin J, Elledge SJ<br>Requirement of ATM-dependent phosphorylation of BRCA1 in the DNA damage response to double-strand breaks<br>SCIENCE. 1999 NOV 5; 286 (5442): 1162-1166	6	791
31	Meijers-Heijboer H, van den Ouweland A, Klijn J, Wasielewski M, de Snoo A, et al.<br>Low-penetrance susceptibility to breast cancer due to CHEK2*1100delC in noncarriers of BRCA1 or BRCA2 mutations<br>NATURE GENETICS. 2002 MAY; 31 (1): 55-59	0	775
32	Scully R, Chen JJ, Ochs RL, Keegan K, Hoekstra M, et al.<br>Dynamic changes of BRCA1 subnuclear location and phosphorylation state are initiated by DNA damage<br>CELL. 1997 AUG 8; 90 (3): 425-435	14	758
33	Kuchenbaecker KB, Hopper JL, Barnes DR, Phillips KA, Mooij TM, et al.<br>Risks of Breast, Ovarian, and Contralateral Breast Cancer for BRCA1 and BRCA2 Mutation Carriers<br>JAMA-JOURNAL OF THE AMERICAN MEDICAL ASSOCIATION. 2017 JUN 20; 317 (23): 2402-2416	0	726
34	Risch HA, McLaughlin JR, Cole DEC, Rosen B, Bradley L, et al.<br>Prevalence and penetrance of germline BRCA1 and BRCA2 mutations in a population series of 649 women with ovarian cancer	9	720

	AMERICAN JOURNAL OF HUMAN GENETICS. 2001 MAR; 68 (3): 700-710		
35	Jonkers J, Meuwissen R, van der Gulden H, Peterse H, van der Valk M, et al.<br>Synergistic tumor suppressor activity of BRCA2 and p53 in a conditional mouse model for breast cancer<br>NATURE GENETICS. 2001 DEC; 29 (4): 418-425	4	720
36	Burke W, Daly M, Garber J, Botkin J, Kahn MJE, et al.<br>Recommendations for follow-up care of individuals with an inherited predisposition to cancer .2. BRCA1 and BRCA2<br>JAMA-JOURNAL OF THE AMERICAN MEDICAL ASSOCIATION. 1997 MAR 26; 277 (12): 997-1003	5	717
37	Rebbeck TR, Friebel T, Lynch HT, Neuhausen SL, van't Veer L, et al.<br>Bilateral prophylactic mastectomy reduces breast cancer risk in BRCA1 and BRCA2 mutation carriers: The PROSE study group<br>JOURNAL OF CLINICAL ONCOLOGY. 2004 MAR 15; 22 (6): 1055-1062	3	717
38	Foulkes WD, Stefansson IM, Chappuis PO, Begin LR, Goffin JR, et al.<br>Germline BRCA1 mutations and a basal epithelial phenotype in breast cancer<br>JOURNAL OF THE NATIONAL CANCER INSTITUTE. 2003 OCT 1; 95 (19): 1482-1485	4	705
39	Thompson D, Easton DF<br>Cancer incidence in BRCA1 mutation carriers<br>JNCI-JOURNAL OF THE NATIONAL CANCER INSTITUTE. 2002 SEP 18; 94 (18): 1358-1365	1	693
40	Moynahan ME, Pierce AJ, Jasin M<br>BRCA2 is required for homology-directed repair of chromosomal breaks<br>MOLECULAR CELL. 2001 FEB; 7 (2): 263-272	13	684
41	Roy R, Chun J, Powell SN<br>BRCA1 and BRCA2: different roles in a common pathway of genome protection<br>NATURE REVIEWS CANCER. 2012 JAN; 12 (1): 68-78	0	671
42	Xu XL, Weaver Z, Linke SP, Li CL, Gotay J, et al.<br>Centrosome amplification and a defective G(2)-M cell cycle checkpoint induce genetic instability in BRCA1 exon 11 isoform-deficient cells<br>MOLECULAR CELL. 1999 MAR; 3 (3): 389-395	11	667
43	Tavtigian SV, Simard J, Rommens J, Couch F, ShattuckEidens D, et al.<br>The complete BRCA2 gene and mutations in chromosome 13q-linked kindreds<br>NATURE GENETICS. 1996 MAR; 12 (3): 333-337	15	665
44	Sakai W, Swisher EM, Karlan BY, Agarwal MK, Higgins J, et al.<br>Secondary mutations as a mechanism of cisplatin resistance in BRCA2-mutated cancers<br>NATURE. 2008 FEB 28; 451 (7182): 1116-U9	4	662
45	Edwards SL, Brough R, Lord CJ, Natrajan R, Vatcheva R, et al.<br>Resistance to therapy caused by intragenic deletion in BRCA2<br>NATURE. 2008 FEB 28; 451 (7182): 1111-U8	5	661
46	Rahman N, Seal S, Thompson D, Kelly P, Renwick A, et al.<br>PALB2, which encodes a BRCA2-interacting protein, is a breast cancer susceptibility gene<br>NATURE GENETICS. 2007 FEB; 39 (2): 165-167	0	659

47	Meijers-Heijboer H, van Geel B, van Putten WLJ, Henzen-Logmans SC, Seynaeve C, et al.<br>Breast cancer after prophylactic bilateral mastectomy in women with a BRCA1 or BRCA2 mutation.<br>NEW ENGLAND JOURNAL OF MEDICINE. 2001 JUL 19; 345 (3): 159-164	0	648
48	Schlacher K, Christ N, Siaud N, Egashira A, Wu H, et al.<br>Double-Strand Break Repair-Independent Role for BRCA2 in Blocking Stalled Replication Fork Degradation by MRE11<br>CELL. 2011 MAY 13; 145 (4): 529-542	2	641
49	Pujade-Lauraine E, Ledermann JA, Selle F, Gebski V, Penson RT, et al.<br>Olaparib tablets as maintenance therapy in patients with platinum-sensitive, relapsed ovarian cancer and a BRCA1/2 mutation (SOLO2/ENGOT-Ov21): a double-blind, randomised, placebo-controlled, phase 3 trial<br>LANCET ONCOLOGY. 2017 SEP; 18 (9): 1274-1284	0	638
50	Lerman C, Narod S, Schulman K, Hughes C, GomezCaminero A, et al.<br>BRCA1 testing in families with hereditary breast-ovarian cancer - A prospective study of patient decision making and outcomes<br>JAMA-JOURNAL OF THE AMERICAN MEDICAL ASSOCIATION. 1996 JUN 26; 275 (24): 1885-1892	2	625
51	Frank TS, Deffenbaugh AM, Reid JE, Hulick M, Ward BE, et al.<br>Clinical characteristics of individuals with germline mutations in BRCA1 and BRCA2: Analysis of 10,000 individuals<br>JOURNAL OF CLINICAL ONCOLOGY. 2002 MAR 15; 20 (6): 1480-1490	3	625
52	Rottenberg S, Jaspers JE, Kersbergen A, van der Burg E, Nygren AOH, et al.<br>High sensitivity of BRCA1-deficient mammary tumors to the PARP inhibitor AZD2281 alone and in combination with platinum drugs<br>PROCEEDINGS OF THE NATIONAL ACADEMY OF SCIENCES OF THE UNITED STATES OF AMERICA. 2008 NOV 4; 105 (44): 17079-17084	2	618
53	Narod SA, Foulkes WD<br>BRCA1 and BRCA2: 1994 and beyond<br>NATURE REVIEWS CANCER. 2004 SEP; 4 (9): 665-676	3	607
54	Bouwman P, Aly A, Escandell JM, Pieterse M, Bartkova J, et al.<br>53BP1 loss rescues BRCA1 deficiency and is associated with triple-negative and BRCA-mutated breast cancers<br>NATURE STRUCTURAL & MOLECULAR BIOLOGY. 2010 JUN; 17 (6): 688-U56	1	606
55	Peto J, Collins N, Barfoot R, Seal S, Warren W, et al.<br>Prevalence of BRCA1 and BRCA2 gene mutations in patients with early-onset breast cancer<br>JOURNAL OF THE NATIONAL CANCER INSTITUTE. 1999 JUN 2; 91 (11): 943-949	5	603
56	Lakhani SR, van de Vijver MJ, Jacquemier J, Anderson TJ, Osin PP, et al.<br>The pathology of familial breast cancer: Predictive value of immunohistochemical markers estrogen receptor, progesterone receptor, HER-2, and p53 in patients with mutations in BRCA1 and BRCA2<br>JOURNAL OF CLINICAL ONCOLOGY. 2002 MAY 1; 20 (9): 2310-2318	4	593
57	Wu LJC, Wang ZW, Tsan JT, Spillman MA, Phung A, et al.<br>Identification of a RING protein that can interact in vivo with the BRCA1 gene product<br>NATURE GENETICS. 1996 DEC; 14 (4): 430-440	10	567

58	Friedman LS, Ostermeyer EA, Szabo CI, Dowd P, Lynch ED, et al.<br>Confirmation Of BRCA1 Lay Analysis Of Germline Mutations Linked To Breast And Ovarian-Cancer In 10 Families<br>NATURE GENETICS. 1994 DEC; 8 (4): 399-404	12	553
59	Xu XL, Wagner KU, Larson D, Weaver Z, Li CL, et al.<br>Conditional mutation of Brca1 in mammary epithelial cells results in blunted ductal morphogenesis and tumour formation<br>NATURE GENETICS. 1999 MAY; 22 (1): 37-43	5	553
60	Roa BB, Boyd AA, Volcik K, Richards CS<br>Ashkenazi Jewish population frequencies for common mutations in BRCA1 and BRCA2<br>NATURE GENETICS. 1996 OCT; 14 (2): 185-187	6	544
61	Hakem R, delaPompa JL, Sirard C, Mo R, Woo M, et al.<br>The tumor suppressor gene Brca1 is required for embryonic cellular proliferation in the mouse<br>CELL. 1996 JUN 28; 85 (7): 1009-1023	16	538
62	Rebbeck TR, Kauff ND, Domchek SM<br>Meta-analysis of Risk Reduction Estimates Associated With Risk-Reducing Salpingo-oophorectomy in BRCA1 or BRCA2 Mutation Carriers<br>JNCI-JOURNAL OF THE NATIONAL CANCER INSTITUTE. 2009 JAN 21; 101 (2): 80-87	1	530
63	Couch FJ, DeShano ML, Blackwood MA, Calzone K, Stopfer J, et al.<br>BRCA1 mutations in women attending clinics that evaluate the risk of breast cancer<br>NEW ENGLAND JOURNAL OF MEDICINE. 1997 MAY 15; 336 (20): 1409-1415	2	529
64	Hompson ME, Jensen RA, Obermiller PS, Page DL, Holt JT<br>Decreased Expression Of BRCA1 Accelerates Growth And Is Often Present During Sporadic Breast-Cancer Progression<br>NATURE GENETICS. 1995 APR; 9 (4): 444-450	5	528
65	Struewing JP, Abeliovich D, Peretz T, Avishai N, Kaback Mm, et al.<br>The Carrier Frequency Of The BRCA1 185Delag Mutation Is Approximately 1 Percent In Ashkenazi-Jewish Individuals<br>NATURE GENETICS. 1995 OCT; 11 (2): 198-200	5	528
66	Xia B, Sheng Q, Nakanishi K, Ohashi A, Wu JM, et al.<br>Control of BRCA2 cellular and clinical functions by a nuclear partner, PALB2<br>MOLECULAR CELL. 2006 JUN 23; 22 (6): 719-729	2	521
67	Wang B, Matsuoka S, Ballif BA, Zhang D, Smogorzewska A, et al.<br>Abraxas and RAP80 form a BRCA1 protein complex required for the DNA damage response<br>SCIENCE. 2007 MAY 25; 316 (5828): 1194-1198	1	512
68	Escribano-Diaz C, Orthwein A, Fradet-Turcotte A, Xing MT, Young JTF, et al.<br>A Cell Cycle-Dependent Regulatory Circuit Composed of 53BP1-RIF1 and BRCA1-CtIP Controls DNA Repair Pathway Choice<br>MOLECULAR CELL. 2013 MAR 7; 49 (5): 872-883	0	511
69	King MC, Wieand S, Hale K, Lee M, Walsh T, et al.<br>Tamoxifen and breast cancer incidence among women with inherited mutations in BRCA1	4	509

	and BRCA2 - National Surgical Adjuvant Breast and Bowel Project (NSABP-P1) Breast Cancer Prevention Trial<br>JAMA-JOURNAL OF THE AMERICAN MEDICAL ASSOCIATION. 2001 NOV 14; 286 (18): 2251-2256		
70	Lakhani SR, Jacquemier J, Sloane JP, Gusterson BA, Anderson TJ, et al.<br>Multifactorial analysis of differences between sporadic breast cancers and cancers involving BRCA1 and BRCA2 mutations<br>JNCI-JOURNAL OF THE NATIONAL CANCER INSTITUTE. 1998 AUG 5; 90 (15): 1138-1145	6	508
71	Sobhian B, Shao GZ, Lilli DR, Culhane AC, Moreau LA, et al.<br>RAP80 targets BRCA1 to specific ubiquitin structures at DNA damage sites<br>SCIENCE. 2007 MAY 25; 316 (5828): 1198-1202	1	507
72	Parmigiani G, Berry DA, Aguilar O<br>Determining carrier probabilities for breast cancer-susceptibility genes BRCA1 and BRCA2<br>AMERICAN JOURNAL OF HUMAN GENETICS. 1998 JAN; 62 (1): 145-158	1	503
73	Scully R, Livingston DM<br>In search of the tumour-suppressor functions of BRCA1 and BRCA2<br>NATURE. 2000 NOV 23; 408 (6811): 429-432	6	500
74	Jensen DE, Proctor M, Marquis ST, Gardner HP, Ha SI, et al.<br>BAP1: a novel ubiquitin hydrolase which binds to the BRCA1 RING finger and enhances BRCA1-mediated cell growth suppression<br>ONCOGENE. 1998 MAR 5; 16 (9): 1097-1112	0	494
75	Patel KJ, Yu VPCC, Lee HS, Corcoran A, Thistlethwaite FC, et al.<br>Involvement of Brca2 in DNA repair<br>MOLECULAR CELL. 1998 FEB; 1 (3): 347-357	16	492
76	Pal T, Permuth-Wey J, Betts JA, Krischer JP, Fiorica J, et al.<br>BRCA1 and BRCA2 mutations account for a large proportion of ovarian carcinoma cases<br>CANCER. 2005 DEC 15; 104 (12): 2807-2816	2	492
77	Schlacher K, Wu H, Jasin M<br>A Distinct Replication Fork Protection Pathway Connects Fanconi Anemia Tumor Suppressors to RAD51-BRCA1/2<br>CANCER CELL. 2012 JUL 10; 22 (1): 106-116	0	491
78	Zhong Q, Chen CF, Li S, Chen YM, Wang CC, et al.<br>Association of BRCA1 with the hRad50-hMre11-p95 complex and the DNA damage response<br>SCIENCE. 1999 JUL 30; 285 (5428): 747-750	7	490
79	Davies AA, Masson JY, Mcllwraith MJ, Stasiak AZ, Stasiak A, et al.<br>Role of BRCA2 in control of the RAD51 recombination and DNA repair protein<br>MOLECULAR CELL. 2001 FEB; 7 (2): 273-282	7	488
80	Hashizume R, Fukuda M, Maeda I, Nishikawa H, Oyake D, et al.<br>The RING heterodimer BRCA1-BARD1 is a ubiquitin ligase inactivated by a breast cancer-derived mutation<br>JOURNAL OF BIOLOGICAL CHEMISTRY. 2001 MAY 4; 276 (18): 14537-14540	3	487
81	Yang HJ, Jeffrey PD, Miller J, Kinnucan E, Sun YT, et al.	3	486

	BRCA2 function in DNA binding and recombination from a BRCA2-DSS1-ssDNA structure<br>SCIENCE. 2002 SEP 13; 297 (5588): 1837-1848		
82	Mavaddat N, Peock S, Frost D, Ellis S, Platte R, et al.<br>Cancer Risks for BRCA1 and BRCA2 Mutation Carriers: Results From Prospective Analysis of EMBRACE<br>JNCI-JOURNAL OF THE NATIONAL CANCER INSTITUTE. 2013 JUN; 105 (11): 812-822	1	483
83	Harkin DP, Bean JM, Miklos D, Song YH, Truong VB, et al.<br>Induction of GADD45 and JNK/SAPK-dependent apoptosis following inducible expression of BRCA1<br>CELL. 1999 MAY 28; 97 (5): 575-586	1	481
84	Cantor SB, Bell DW, Ganesan S, Kass EM, Drapkin R, et al.<br>BACH1, a novel helicase-like protein, interacts directly with BRCA1 and contributes to its DNA repair function<br>CELL. 2001 APR 6; 105 (1): 149-160	5	480
85	Chen JJ, Silver DP, Walpita D, Cantor SB, Gazdar AF, et al.<br>Stable interaction between the products of the BRCA1 and BRCA2 tumor suppressor genes in mitotic and meiotic cells<br>MOLECULAR CELL. 1998 SEP; 2 (3): 317-328	14	477
86	Pellegrini L, Yu DS, Lo T, Anand S, Lee M, et al.<br>Insights into DNA recombination from the structure of a RAD51-BRCA2 complex<br>NATURE. 2002 NOV 21; 420 (6913): 287-293	2	475
87	Goggins M, Schutte M, Lu J, Moskaluk CA, Weinstein CL, et al.<br>Germline BRCA2 gene mutations in patients with apparently sporadic pancreatic carcinomas<br>CANCER RESEARCH. 1996 DEC 1; 56 (23): 5360-5364	10	474
88	Hartmann LC, Sellers TA, Schaid DJ, Frank TS, Soderberg CL, et al.<br>Efficacy of bilateral prophylactic mastectomy in BRCA1 and BRCA2 gene mutation carriers<br>JNCI-JOURNAL OF THE NATIONAL CANCER INSTITUTE. 2001 NOV 7; 93 (21): 1633-1637	3	470
89	Lakhani SR, Reis-Filho JS, Fulford L, Penault-Llorca F, van der Vjiver M, et al.<br>Prediction of BRCA1 status in patients with breast cancer using estrogen receptor and basal phenotype<br>CLINICAL CANCER RESEARCH. 2005 JUL 15; 11 (14): 5175-5180	0	470
90	Tavtigian SV, Deffenbaugh AM, Yin L, Judkins T, Scholl T, et al.<br>Comprehensive statistical study of 452 BRCA1 missense substitutions with classification of eight recurrent substitutions as neutral<br>JOURNAL OF MEDICAL GENETICS. 2006 APR; 43 (4): 295-305	0	464
91	Brose MS, Rebbeck TR, Calzone KA, Stopfer JE, Nathanson KL, et al.<br>Cancer risk estimates for BRCA1 mutation carriers identified in a risk evaluation program<br>JOURNAL OF THE NATIONAL CANCER INSTITUTE. 2002 SEP 18; 94 (18): 1365-1372	3	459

92	17 Callebaut I, Mornon JP<br>From BRCA1 to RAP1: A widespread BRCT module closely associated with DNA repair<br>FEBS LETTERS. 1997 JAN 2; 400 (1): 25-30	4	457
93	25 Somasundaram K, Zhang HB, Zeng YX, Houvras Y, Peng Y, et al.<br>Arrest of the cell cycle by the tumour-suppressor BRCA1 requires the CDK-inhibitor p21(WAF1/CiP1)<br>NATURE. 1997 SEP 11; 389 (6647): 187-190	4	455
94	Wright MH, Calcagno AM, Salcido CD, Carlson MD, Ambudkar SV, et al.<br>Brca1 breast tumors contain distinct CD44(+)/CD24(-) and CD133(+) cells with cancer stem cell characteristics<br>BREAST CANCER RESEARCH. 2008; 10 (1): Art. No. R10	0	451
95	Walsh T, Casadei S, Coats KH, Swisher E, Stray SM, et al.<br>Spectrum of mutations in BRCA1, BRCA2, CHEK2, and TP53 in families at high risk of breast cancer<br>JAMA-JOURNAL OF THE AMERICAN MEDICAL ASSOCIATION. 2006 MAR 22; 295 (12): 1379-1388	0	449
96	Bhattacharyya A, Ear US, Koller BH, Weichselbaum RR, Bishop DK<br>The breast cancer susceptibility gene BRCA1 is required for subnuclear assembly of Rad51 and survival following treatment with the DNA cross-linking agent cisplatin<br>JOURNAL OF BIOLOGICAL CHEMISTRY. 2000 AUG 4; 275 (31): 23899-23903	4	448
97	Risch HA, McLaughlin JR, Cole DEC, Rosen B, Bradley L, et al.<br>Population BRCA1 and BRCA2 mutation frequencies and cancer penetrances: A kin-cohort study in Ontario, Canada<br>JNCI-JOURNAL OF THE NATIONAL CANCER INSTITUTE. 2006 DEC 6; 98 (23): 1694-1706	0	445
98	Ford D, Easton DF, Peto J<br>Estimates Of The Gene-Frequency Of BRCA1 And Its Contribution To Breast And Ovarian-Cancer Incidence<br>AMERICAN JOURNAL OF HUMAN GENETICS. 1995 DEC; 57 (6): 1457-1462	5	443
99	23 Lakhani SR, Easton DF, Stratton MR, StorferIsser A, Anderson TJ, et al.<br>Pathology of familial breast cancer: Differences between breast cancers in carriers of BRCA1 or BRCA2 mutations and sporadic cases<br>LANCET. 1997 MAY 24; 349 (9064): 1505-1510	6	443
100	Hacia JG, Brody LC, Chee MS, Fodor SPA, Collins FS<br>Detection of heterozygous mutations in BRCA1 using high density oligonucleotide arrays and two-colour fluorescence analysis<br>NATURE GENETICS. 1996 DEC; 14 (4): 441-447	0	441
